# Practical Notes and Formulæ

**Published:** 1884-08-20

**Authors:** 


### PRACTICAL NOTES AND FORMULÆ.

**The Administration of Cotoine.**—[Obtained from coto bark, a South Ameriean tree.]—Gielt considered coto as a specific against diarrhœa, and he was the first to utilize it in Europe. He gave it in powder, in doses of gr. vijss, four or six times a day, and in tincture (one part of the bark to nine of alcohol at 85°) in doses of gtts. x every two hours. Parsons, Fronmuller, Yeo and Rohrer have used the following preparations with advantage:

℞ Fluid ext. of coto ............................gtts. vj.
Compound tinct. of cardamom .. ...........gtts. lx.
M. and triturate with
Mucilage of acacia ..........................fl. ʒ iij,
And add
Simple syrup ............ ...................fl. ʒ ij,
Water .......................................fl. ℥ vj.
S. A tablespoonful (Burney Yeo).

℞ Cotoine.....................................gr. j-jss,
Alcohol (rectified)..........................fl. ʒ ijss,
Syrup.......................................fl. ℥ j,
Distilled water.............................fl. ℥ iv.
M. S. A dessertspoonful every hour (Burkhart).

℞ Cotoine.....................................gr. jvss.
Divide into three papers. To be taken in wafers.

℞ Cotoine.....................................gr. jvss,
Gum emulsin.................................fl. ℥ vj.
M. To be taken four times a day (Albertoni).

℞ Cotoine.....................................gr. vj,
Pure cotoine................................gr. xv,
Subnitrate bismuth........... .............ʒ iv,
Mucilage....................................fl. ℥ vj.
M. S. For deep injection every half hour or every hour.

Burkhart and Jobst, who highly recommended the last in cholera, recommended the addition of fl. ʒ ss to fl. ʒ j of chloral to alleviate the pains, and an increase in the dose according to the gravity of the disease, the preparations of coto having no narcotic or other bad action.

*Therapy*.—Cotoine and paracotoine are chiefly used in diarrhœa (without intestinal ulceration), complicating the different forms of mental alienation, phthisis, pillagea, etc. It is contraindicated in the catarrh of alcoholics and those suffering from cirrhosis. They have been used in cholera, in the night sweats of phthisis, in infantile diarrhœa, and in sialorrhœa. Riggi believes that cotoine is sometimes indicated in chronic intestinal catarrh.—*Le Progres Med.; Amer. Jour. Med. Sci.; Drug. Cir.*

**No. 3. Elixir Ammonii Valerianatis.**—[Elixir of Valerianate of Ammonium.—

| | | |
|---|---|---|
| ℞ | Valerianate of ammonium | 256 grains, |
| | Chloroform | 6 minims, |
| | Tincture of Vanilla (U. S. P.) | 2 fl. ʒ, |
| | Water of ammonia (10 p. ct.) a sufficient quantity, | |
| | Tincture of cudbear (No. 78) | 2 fl. drachms, |
| | Simple elixir (No. 46), enough to make | 16 fl. ounces. |

Dissolve the valerianate of ammonium in about 12 fluid ounces of simple elixir, in an open graduated vessel, and add water of ammonia, in drops, until a faint excess of ammonia is perceptible in the liquid. Then add the chloroform, tincture of vanilla and tincture of cudbear, and, finally, enough simple elixir to make 16 fluid ounces.

*Note.*—Should the odor of valerianic acid become perceptible after the elixir has been kept for some time, it may be overcome by cautious supersaturation with water of ammonia.—*Druggist's Circular.*

**Tartaric Acid in Diphtheria.**—Prof. Vidal, of Paris, employs the following in diphtheria, and claims for it an almost specific action:

| | | |
|---|---|---|
| ℞ | Tartaric acid | ʒ ijss, |
| | Glycerine | ℥ ss, |
| | Peppermint water | ʒ vij |
| M. | | |

A brush dipped in this solution is made to touch, every three hours, the diphtheritic patches, which soon reduces them to a pulpy, liquid mass, which is afterward easily removed. In the interval of the applications of the tartaric acid the false membranes are touched with citron juice.—*Med. and Surg. Rep.*

**Loomis' Tonic Bitters.**—

| | | |
|---|---|---|
| ℞ | Quiniæ sulphat | grs. xxx, |
| | Acidi sulph. dil | q. s., |
| | Aquæ | fl ℥ ij, |
| | Tinct. ferri chlor | fl. ℥ i-ij, |
| | Spts. chloroformi | fl. ʒ vj, |
| | Glycerinæ q. s. ad | fl. ℥ jv. |

Dissolve the quinine in the water by aid of the acid, and add the other ingredients.—*Nat. Drug.*

**Walnut Hair Dye** (Barber, Dexter, Mo.)—To the expressed juice of green walnut shells add ten per cent. of alcohol, let stand ten days and filter. Before applying it, wash the hair with a solution of sal soda or salts of tartar. One application of the dye, wetting the hair thoroughly, will usually suffice to produce a dark brown or black color.—*Nat. Drug.*

**Diarrhœa and Dysentery of Children.**—

℞ Monobrominated Camph ....................gr. jv,

Sugar of milk..............................gr. xl,

Pepsin,

Subnitrate bismuth } aa........................ʒ j.

Triturate and divide into powders No. 22.

Take one every three hours until the evacuations are somewhat restrained, and then one to be given after each evacuation until relieved. An opiate may be added or not according to circumstances. Usually it is better to omit the opium, as it tends to lock the upper bowel and derange the secretions. If dysenteric symptoms exist, five grains of chalk and mercury added once a day will be found an efficient auxiliary.

**For Diarrhœa and Dysentery.**—

R Benzoate of soda...............................ʒ j,
Simple elixir.....................................℥ ij.
M. Dose for an adult two teaspoonfuls every half to one hour.

May be combined or alternated with bismuth or opiates.

**Dysmenorrhœa.**—This is another very painful affliction with which many females suffer, and many recipes are given in our medical journals. I will append a few, not specially new, but good and reliable with me.

℞ Fl. ext. cimicifuga...............................ʒ j,
Fl. ext. pulsatilla (anemone).......................ʒ j,
Aqua.............................................℥ jv.
M. A teaspoonful three or four times a day, commencing a few days in advance of the expected crisis. If this fails, add ʒ ij of fl. ext. gelsemium and give in like manner.

Another formula is this:

℞ Fl. ext. viburnum op.............................℥ j,
Fl. ext. gelsemium.................................ʒ j,
Aqua, q. s. ft.....................................℥ ij.
M. Thirty gtts. every two or three hours, anticipating the attack a few hours if possible.

One of these recipes have always brought relief to my patients; and a tonic course in the interval, as indicated, iron or senecio in some combination, will, in a few months, overcome the trouble.—*Med. Summary.*

**Diarrhœa in Children.**—

℞ Zinci oxidi.....................................gr. j,

Bismuth subnit...................................ʒ j,

Pepsin pulverized................................grs. x,

Sacch. lactis....................................q. s.

M. ft. Pul. No. xii. Sig. One every three or four hours.

